# Common carotid artery occlusion during slaughter without stunning in cattle: incidence and evidence for physiological significance—a systematic review

**DOI:** 10.3389/fvets.2026.1868851

**Published:** 2026-09-15

**Authors:** Elie Girsowicz, Ari Z. Zivotofsky, Stuart D. Rosen, Jacob R. Hascalovici

**Affiliations:** 1 Department of Surgery, McGill University, Montreal, QC, Canada; 2 Gonda Brain Center, Bar Ilan University, Ramat Gan, Israel; 3 National Heart and Lung Institute, Imperial College, London, United Kingdom; 4 Albert Einstein College of Medicine, Bronx, NY, United States; 5 Hackensack University School of Medicine, Nutley, NJ, United States

**Keywords:** carotid occlusions, common carotid arteries, consciousness, cortex, false aneurysm, halal slaughter, ruminants, shechita

## Abstract

**Introduction:**

Slaughter by exsanguination via ventral neck incision (SEVNI), as performed in religious slaughter, has been criticized on the grounds that carotid occlusion may impair exsanguination and potentially delay loss of consciousness (LOC), leading to unnecessary pain and distress. The objective of this review is to evaluate the literature specifically addressing the prevalence of carotid occlusions (CO) in SEVNI of cattle and the available evidence for their potential consequences on cerebral perfusion, cortical function, and inferred LOC.

**Methods:**

A systematic search of PubMed, Google Scholar, Cochrane Library, Medline, and Web of Science identified studies assessing the prevalence and significance of CO in cattle after SEVNI. Non-cattle studies, reviews, commentaries and abstracts without full text were excluded. Studies were independently reviewed by co-authors to minimize risk of bias. Due to heterogeneity in methods and outcomes, results were synthesized narratively.

**Results:**

Fifteen studies met the inclusion criteria. ROBINS-I framework determined the overall risk of bias to be moderate. Across eligible prevalence cohorts, 13.8% of animals exhibited at least one reported carotid occlusive phenomenon. Conclusions suggesting delayed LOC in animals with CO are primarily inferential and rely on indirect evidence from a small number of studies with methodological limitations.

**Discussion:**

Based on the available evidence, while CO do appear to occur in a subgroup of animals following SEVNI, there is insufficient evidence to demonstrate that they preserve cerebral perfusion or sustain cortical function at levels sufficient to delay LOC as inferred from available proxy measures. Available physiological and mathematical modeling data instead suggest that cortical perfusion would be expected to fall rapidly after bilateral carotid transection, but these data do not directly measure consciousness.

**Systematic review registration:**

https://www.crd.york.ac.uk/PROSPERO/view/CRD420261325822, identifier PROSPERA; (registration number CRD420261325822).

## Introduction

Debate surrounding slaughter without prior stunning, including Jewish *shechita* and Muslim halal slaughter (*dhabīhah*), remains one of the most enduring issues in animal welfare science (1–6) and policy (7–11). From an animal welfare perspective, debate has centered on whether slaughter without stunning reliably produces rapid loss of consciousness (LOC) and insensibility, concerns that have been cited in policy discussions and legislative efforts to restrict the practice (12). Several countries, including Denmark, Sweden, Norway, Iceland, Switzerland, New Zealand and parts of Belgium prohibit all slaughter of cattle without stunning, citing welfare concerns. Other jurisdictions permit slaughter without stunning exclusively for religious purposes, including the United Kingdom, parts of the European Union under specific exemptions, the United States, Canada, Israel and Australia. At the core of this debate is the question of animal pain and suffering at the time of slaughter and immediately post-cut (13–15). Proponents of preslaughter stunning maintain that mechanical (e.g., captive bolt) or electrical stunning renders the animal immediately unconscious and insensible, thereby minimizing suffering and representing the most humane method available (3, 16, 17). Conversely, proponents of slaughter without stunning contend that slaughter by exsanguination via ventral neck incision (SEVNI) results in a near-instantaneous loss of cerebral blood flow and arterial pressure, leading to immediate cessation of integrated cortical function and, therefore, LOC and insensibility (18–20).

Concerns regarding slaughter without stunning largely rest on the hypothesis that LOC following SEVNI is progressive rather than immediate. In their methodological review, Terlouw et al. characterize bleeding without stunning as producing “a global progressive dysfunction of cortical and subcortical structures including the reticular formation” and further explain that “Widespread anoxia in the brain following bleeding of non-stunned animals results also in a global progressive dysfunction of cortical and subcortical structures…” (4). Similarly, Johnson et al. describe a temporal unfolding of this process, referring to “the progression of this salient cerebral process in non-stunned animals at slaughter” (2) and stating unequivocally that “It is clear that slaughter by neck incision does not render ruminants immediately insensible.” Together, these descriptions consistently frame LOC after exsanguination as a progressive deterioration of cerebral function, characterized by staged loss of cortical function preceding complete insensibility.

Two principal mechanisms have been proposed to account for this extended, progressive process despite the interruption of the cerebral blood supply due to the complete severing of the common carotid arteries. The first is the vertebral artery hypothesis, which posits that the vertebral arteries, unsevered by ventral neck incision, may continue to perfuse the brain and thereby delay the onset of LOC (21, 22). The second is the carotid occlusion hypothesis which posits that post-severance occlusive phenomena at the cut ends of the common carotid arteries may impede exsanguination, thereby altering cerebral hemodynamics in a manner that delays LOC or permits transient restoration of cerebral perfusion (16, 23–26). A critical but largely unexamined question is whether the timing of carotid occlusion formation is physiologically compatible with influencing LOC in the context of a loss of cerebral blood flow and pressure following SEVNI (27–30).

The present systematic review examines the carotid occlusion hypothesis by assessing the reported incidence of CO in bovines following SEVNI and evaluating whether the available evidence indicates that such occlusions have been shown to alter, preserve, or restore cerebral perfusion and thereby delay inferred LOC. For the purposes of this review, the term *carotid occlusion* (CO) is used as an umbrella term referring to any obstruction of common carotid arterial outflow following severance, including thrombotic clot formation, arterial retraction, and the development of false aneurysms (FA). When discussing individual studies, however, the terminology used by the original authors (e.g., “false aneurysm,” “ballooning,” or clot formation) is retained to preserve fidelity to the source literature. Importantly, the identification of a false aneurysm or other structural abnormality does not necessarily demonstrate functional cessation of carotid blood flow. Accordingly, structural findings and directly observed flow arrest are distinguished where possible throughout this review.

The primary outcome of interest is the potential relationship between CO and LOC; however, because consciousness cannot be directly measured during commercial slaughter, LOC is treated here as an inferred physiological endpoint based on proxy measures of cerebral perfusion, cortical electrical activity, and behavior.

## Methods

A registered (PROSPERA; https://www.crd.york.ac.uk/PROSPERO/view/CRD420261325822) systematic review of the literature was conducted to clarify the incidence and evidence of the functional significance of CO in bovines in the context of SEVNI. PRISMA guidelines for reporting of systematic reviews were followed (31). PubMed, Google Scholar, the Cochrane Library, MEDLINE, and Web of Science were searched using the following search strategy: (calf OR bovine) AND (“carotid occlusion” OR “carotid occlusions” OR “false aneurysm” OR “false aneurysms”) AND (“blood flow” OR “blood pressure”) AND slaughter AND (“cerebral perfusion” OR “cerebral circulation” OR “cortical function”); and (“carotid occlusion” OR “carotid occlusions” OR “false aneurysm” OR “false aneurysms”) AND (calf OR bovine) AND blood pressure AND (shechita OR halal) AND (“cerebral perfusion” OR “cerebral circulation” OR “cortical function”). The last search date was February 25, 2026. To ensure transparency and reproducibility, the complete database-specific search strategies are provided in Supplementary material 1. Eligible study designs included experimental, observational, or physiological studies that reported direct or indirect evaluations or measurements of incidence, hemodynamic effects and evidence of the functional significance of CO in cattle after slaughter without stunning. Exclusion criteria were studies in species other than cattle, studies not measuring CO, reviews or commentaries without original data and abstracts without full text. Reference lists of all the articles obtained were manually reviewed to identify additional relevant studies. Eighty-six articles were screened for relevance. To decrease the risk of bias, the co-authors independently reviewed all the articles. Full texts of 30 potentially eligible studies were assessed for inclusion. Disagreements at any stage were resolved through co-author discussions and consensus. After screening and full-text review, 15 studies met the inclusion criteria (Figure 1). Reference lists of included studies and relevant reviews were also examined to identify additional eligible primary studies, including studies reporting findings that could support a physiological association between carotid occlusion and delayed behavioral, electrophysiological, or hemodynamic endpoints. For each study the authors, year of publication, number of animals included in the study, use of anesthesia and stunning, study design, method of slaughter if applicable, and key findings were extracted and tabulated in Tables 1, 2. Risk of bias for the included studies was assessed using the ROBINS-I framework, a structured tool originally developed for non-randomized intervention studies (32). Because the included evidence consisted of heterogeneous experimental, observational, prevalence, and physiological studies, ROBINS-I was used here as an adapted framework to assess common sources of bias, including confounding, participant selection, intervention classification, deviations from intended interventions, missing data, outcome measurement, and selective reporting. ROBINS-I was used to assess risk of bias within individual studies and was not used to generate a pooled intervention-effect estimate or to determine overall certainty regarding LOC. Data were extracted from five categories of studies: (1) prevalence studies; (2) those employing measures of hemodynamics; (3) cortical electrical activity; (4) behavioral observation; and (5) a computational modeling study. Computational modeling studies were considered separately from experimental studies and were treated as mechanistic, hypothesis-supporting evidence. None of these categories was considered a direct measurement of consciousness. Each was evaluated according to its directness as a proxy for cortical perfusion, integrated cortical function, or behavioral responsiveness. Given substantial heterogeneity in study design and measurement modalities, quantitative pooling and sensitivity analyses were not methodologically appropriate. Therefore, results were synthesized narratively. Prevalence studies were used to estimate how often CO occurs, but not to infer LOC. Hemodynamic studies were used to assess whether CO was associated with preserved or restored blood flow or pressure. Electrophysiological studies were used to assess residual cortical electrical activity, while recognizing that electrical activity is not equivalent to conscious awareness. Behavioral studies were treated as indirect evidence because posture, collapse, and reflexes may persist after cortical LOC. A hemodynamic modeling study provided additional mechanistic support. A formal GRADE rating was not performed because outcomes were mechanistic and not amenable to quantitative pooling. The results of the prevalence studies are summarized (Table 1). Because definitions varied across studies, carotid occlusion was defined inclusively for this descriptive analysis as the occurrence in an animal of at least one reported false aneurysm, carotid occlusion, or observed arrest of carotid blood flow. The descriptive prevalence calculation was restricted to cattle or calves slaughtered without stunning for which animal-level data permitted determination of the presence of at least one such event. The number of affected animals was aggregated across eligible cohorts and divided by the total number of animals in those cohorts. Because of heterogeneity in study design and definitions of carotid occlusion, this calculation represents a descriptive aggregate and not a pooled meta-analytic prevalence estimate. Gregory et al. (25) was retained in the qualitative prevalence review but excluded from the descriptive aggregate for two reasons: the adult cattle were stunned before neck cutting and therefore did not meet the unstunned-cohort criterion, while in the calf cohort stunning status was not clearly specified and animal-level prevalence of at least one carotid ballooning event could not be determined.

**Figure 1:**
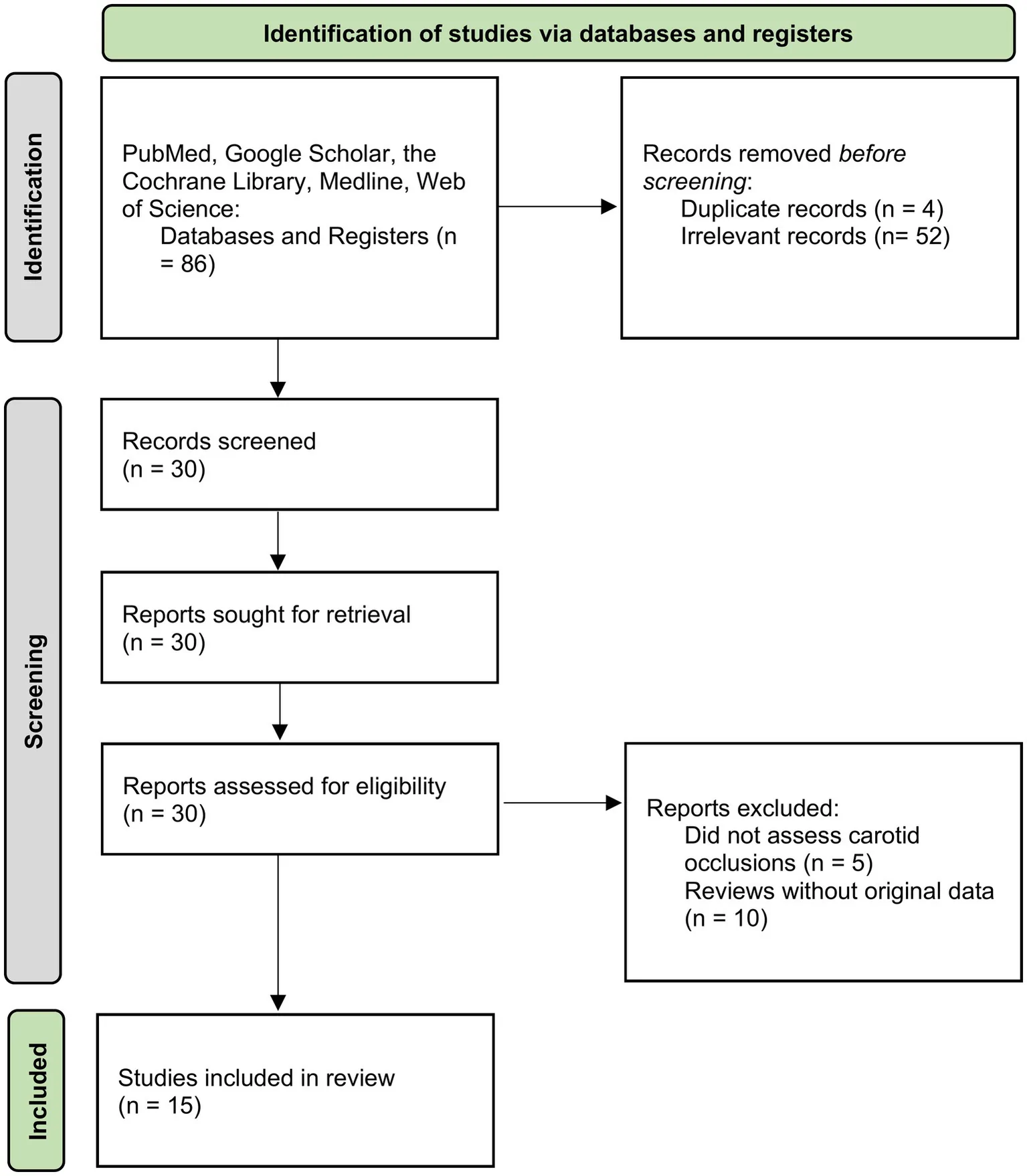
PRISMA flow diagram for study identification, screening, eligibility assessment, and inclusion.

**Table 1:** Prevalence studies reporting carotid occlusion, arrested carotid blood flow or false aneurysm formation after SEVNI in cattle.

Prevalence studies						
Author (Year)	Size (N)	Anesthesia and or Stunning	Study design	Intervention type	Key findings	Value for prevalence calculation
Gregory (25)*	387 Cattle 189 Calves	No anesthesia; captive bolt	Manual palpation	Neck cutting with severance of carotid arteries	16% cattle and 25% calves had CO; 5% cattle and 12% calves had bilateral CO	N/A
Gregory (24)	347 cattle 11 water buffalo	No anesthesia; 20 cattle (control group) electrical stunning with cardiac arrest. No stunning for shechita or halal	Manual palpation	Shechita (*n* = 231) and Halal (cattle *n* = 116; water buffalo *n* = 11)	Shechita and Halal: 1. 10% of all arteries had large CO; 2. 17–18% of cattle had at least one large CO Control group: no CO	62/347
Gregory (34, 35)	126 cattle	No anesthesia; No stunning	Observation	Shechita and Halal	29% of animals exhibited early arrest of carotid blood flow (36/126); approximately 10% had the reported structural/macroscopic occlusion.	36/126
Pozzi (36)	125 cattle	No anesthesia; No stunning	Observation	Shechita	13.6% of arteries had CO. 22.4% of all animals had CO. No association between cut position and CO.	28/125
Supratikno et al. (37)	510 cattle	460 slaughtered with stunning 50 slaughtered without stunning No anesthesia	Post-mortem visual inspection	Halal slaughter	40% showed no false aneurysm (FA), 38% developed unilateral FA, and 22% developed bilateral FA with higher cervical cuts associated with lower FA	30/50

The table summarizes study size, anesthesia or stunning status, study design, slaughter method, and reported frequency of unilateral or bilateral carotid occlusion where available. Bozzo et al. (43) also reported prevalence data but is summarized in Table 2 because its primary analysis evaluated behavioral indicators in relation to false aneurysm formation. *Gregory et al. (25) was retained in the table but excluded from this calculation because the adult cattle were stunned and the calf cohort did not provide both clearly specified stunning status and animal-level prevalence of at least one event. The 13.8% value (255/1,848 animals) is a descriptive aggregate and not a meta-analytic pooled prevalence estimate.

**Table 2:** Studies evaluating the potential functional significance of carotid occlusion after SEVNI.

Author (Year)	Size (N)	Anesthesia and or stunning	Study design	Intervention type	Key findings
Electrophysiology studies					
Bager (39)	21 Calves	General anesthesia; Electrical stunning	ECoG	Electrical head-only stunning followed by recovery Electrical stunning followed by slaughter Slaughter without stunning	Isoelectric point average 49 s. 1 calf from group 3 developed carotid occlusion at 24 s and showed prolonged time to isoelectric.
Anil (40)	Calves: Group 1: *n* = 15 Group 2: *n* = 8	Group 1: General anesthesia; no stunning Group 2: No anesthesia; electrical stunning	Systemic blood pressure measurements and EP on ECoG	Neck Sticking	6/15 calves (40%) developed CO and showed transient increase in BP. Also noted persistent EP and delayed onset of isoelectric ECoG in animals with CO
Anil (23)	20 Calves	General anesthesia; Electrical Stunning	ECoG	Neck sticking *n* = 8 Chest sticking *n* = 12	Neck sticking with CO prolonged time to isoelectric ECoG and persistent EPs Chest sticking no CO
Gibson (14, 15, 41)	7 Calves	General anesthesia; Captive bolt stunning	EEG activity Blood pressure	SEVNI followed by captive bolt after 5 s	CO in 3 animals did not significantly modify femoral artery blood pressure decline or suppression of EEG activity.
Behavior observation					
Blackmore (16)	5 calves 1 young bull	Conscious at exsanguination No stunning	Observation	Bilateral severance of exteriorized carotid arteries and jugular veins (*n* = 4) SEVNI (*n* = 2)	2 calves with CO exhibited extended coordinated attempts to rise (355 and 385 s) when compared to other calves in the study
Gibson (42)	644 cattle	No anesthesia; No stunning	Observation	Halal Low cut: *n* = 561 High cut: *n* = 83	High cut significantly reduced mean time to collapse vs. low neck cut (13.5 ± 1.3 s vs. 18.9 ± 1.1 s; *p* < 0.01) CO score did not independently predict collapse times >60 s Collapse times >20 s was significantly associated with higher CO scores
Bozzo (43)*	1,200 cattle	No anesthesia; No stunning	Observation	Halal at C1 Halal at C2 Halal at C3-4	Overall prevalence of CO: 8.25%; High cut: 7.25% Low cut: 10.25% 37.5% of animals with rhythmic breathing at 90 s had CO
Hemodynamic studies					
Bager (38)	12 Calves	No anesthesia Electrical stunning	Measured jugular venous blood to estimate cerebral blood flow	SEVNI	10 calves without occlusions averaged 4.8% of normal jugular venous flow 2 calves with occlusions had 22 and 39%.
Gregory (26)	174 Cattle	No anesthesia; No stunning	Behavior observation: time to collapse	Halal	8% of animals required >60 s to collapse. Prolonged collapse was significantly associated with presence of CO.
Computational modeling					
Benchimol (27)	N/A	N/A	Numerical Modeling	Bilateral carotid severance	99% reduction in arterial cerebral blood flow in milliseconds. 80% occlusion of all four cut carotid ends: cerebral flow ~8% of presectioning flow.

Included evidence domains include hemodynamic studies, electrophysiological studies, behavioral observation studies, and computational modeling. The table summarizes study design, intervention type, and key findings relevant to cerebral perfusion, cortical electrical activity, behavioral indicators, or inferred LOC. EPs, evoked potentials; ECoG, electrocorticography; EEG, electroencephalography; SEVNI, slaughter by exsanguination via ventral neck incision; CO, carotid occlusion. *Value for prevalence calculation: 99/1,200.

The results of all other studies are summarized (Table 2). Certainty in the body of evidence for each outcome domain was assessed qualitatively using a structured, domain-based approach adapted from GRADE principles, considering risk of bias, consistency, directness, biological plausibility, and coherence across studies. Overall certainty was downgraded for indirectness when studies did not directly measure consciousness, cortical perfusion, or integrated cortical function; for inconsistency when findings differed across study designs or endpoints; and for risk of bias when outcomes depended on behavioral proxies, anesthesia/stunning conditions, or incomplete standardization of slaughter technique (Table 3).

**Table 3:** Certainty and directness of evidence by outcome domain.

Evidence domain	What it supports	Directness of LOC	Certainty
Prevalence studies	CO occur in a subgroup of cattle	Low	Moderate for incidence
Hemodynamic studies	CO may alter residual flow/pressure	Moderate	Low–moderate
Electrophysiology	Residual cortical electrical activity	Moderate	Low
Behavioral studies	Collapse/reflex timing	Low	Low
Modeling	Physiological plausibility	Low–moderate	Low

Evidence categories were rated according to directness for inferring loss of consciousness, risk of bias, consistency, and biological plausibility.

## Results

### Prevalence studies

Gregory (25) conducted a prevalence study of 387 cattle and 189 bobby calves undergoing commercial slaughter with carotid severance, reporting common carotid artery ballooning (≥3 cm) in 16% of cattle and 25% of calves (25).

Gregory (24) investigated the prevalence of CO in 231 cattle slaughtered by shechita, 116 cattle and 11 water buffalo slaughtered by halal methods, and 20 cattle electrically stunned to cardiac arrest prior to bleeding. Large false aneurysms (≥3 cm) developed in approximately 10% of all common carotid arteries during shechita and halal slaughter, with 17–18% of cattle exhibiting at least one large aneurysm and 7–8% showing bilateral aneurysms, whereas no aneurysms occurred in cattle stunned to cardiac arrest before exsanguination (33).

Gregory et al. (34, 35) examined 126 cattle slaughtered without stunning and 100 captive-bolt stunned cattle comparing C1 versus more caudal (C2–C4/C3) cutting positions. In the non-stun cohort, early arrest of carotid blood flow occurred in 29% of animals, was strongly associated with false aneurysm formation at the cardiac ends of the severed arteries, and developed on average 21 s after the neck cut. 95% of arteries with arrested flow had aneurysm scores ≥2, and 6% of cattle exhibited bilateral cessation of bleeding within 60 s (34, 35).

Pozzi (36) evaluated 125 bovines undergoing shechita in an inverted rotating restraint pen and reported common carotid artery occlusion in 34 of 250 common carotid arteries (13.6%), affecting 28 of 125 animals (22.4%). No statistically significant association was found between cut position and carotid occlusion (*p* = 0.36) (36).

Supratikno et al. (37) in a field study of 510 cattle slaughtered (460 with non-penetrative pre-slaughter stunning and 50 without stunning), false aneurysm formation at the severed common carotid arteries was evaluated. In cattle slaughtered without stunning, 40% showed no FA, 38% developed unilateral FA, and 22% developed bilateral FA. Higher cervical cuts near tracheal rings 1–2 were associated with lower FA prevalence and more caudal cervical cuts associated with higher rates of unilateral and bilateral occlusion (37).

Across the five eligible unstunned prevalence cohorts, 255 of 1,848 animals (13.8%) exhibited at least one reported carotid occlusive phenomenon according to the inclusive definition used for this descriptive analysis.

### Hemodynamic studies

Bager (38) evaluated jugular venous blood flow in 12 calves following head-only electrical stunning and throat cutting, reporting that in 10 of 12 animals, jugular flow during the first 1–2 min averaged approximately 4.8% of normal cerebral blood flow, consistent with markedly reduced cerebral perfusion. In two calves, spontaneous carotid occlusion due to clot formation was associated with higher jugular flows (22 and 39% of normal). No visual signs of return to consciousness were observed (38).

### Electrophysiology studies

Bager (39) investigated the onset of insensibility in 21 calves subjected to bilateral carotid and jugular severance, with groups undergoing head-only electrical stunning with recovery, stunning followed by slaughter, or slaughter without stunning. In 17 animals, electrocorticography (ECoG) activity became isoelectric at a mean of 49 s after the cut, with high amplitude low frequency (HALF) activity, consistent with LOC, occurring within 10 s. In one unstunned calf, carotid occlusion from clot formation developed ~24 s after incision and was associated with prolonged HALF activity and delayed isoelectric point (11 min 20 s). FFT spectral analysis demonstrated no restoration of higher-frequency activity indicative of sensibility (39).

Anil (40) examined electrically stunned veal calves undergoing neck sticking, comparing chloralose-anesthetized (*n* = 15) and non-anesthetized (*n* = 8) groups, and reported carotid occlusion (“ballooning”) with large clot formation at the severed arterial ends in 6 of 15 anesthetized animals (40%), resulting in impeded hemorrhage. Calves in the ballooning group exhibited transient recovery episodes in systemic arterial blood pressure corresponding to restricted blood outflow. In the most severe cases, carotid occlusion was associated with delayed decline in systemic blood pressure, persistence of evoked potentials on ECoG and delayed attainment of an isoelectric trace compared with non-occluded animals (40).

Anil (23) compared neck sticking (bilateral carotid and jugular severance; *n* = 8) with chest sticking (brachiocephalic trunk severance; *n* = 12) in 20 electrically stunned calves. In neck-stuck calves, carotid occlusion (“ballooning”) was associated with sustained mean arterial blood pressure and prolonged persistence of evoked potentials, and delayed attainment of an isoelectric ECoG. In contrast, chest sticking prevented carotid occlusion, produced a rapid fall in arterial blood pressure (within ~8 s), and limited time to isoelectric ECoG (23).

Gibson examined 7 calves (halothane-anesthesia) undergoing ventral neck incision, followed 5 s later by non-penetrative captive-bolt stunning, to determine whether post-incisional stunning attenuated EEG responses to ventral neck incision and whether carotid occlusion influenced cortical or hemodynamic outcomes. Neck incision produced decreases in arterial blood pressure and, in some animals, transient EEG spectral changes. Subsequent captive-bolt application rapidly suppressed cortical activity in most calves. Although carotid occlusion was observed in several animals, it did not significantly alter blood pressure decline or EEG suppression profiles (41).

### Behavior observation

Blackmore (16) studied behavioral responses in 5 calves and one young bull slaughtered without stunning, using bilateral exteriorized carotid severance in the calves and a transverse ventral neck incision in the bull. Two calves exhibited extended coordinated attempts to rise (355 and 385 s), and both were found to have retracted common carotid arteries occluded by clotted blood (16).

Gregory (26) conducted an observational study of 174 cattle slaughtered without stunning under halal upright restraint and reported that 8% required ≥60 s to achieve final collapse, with prolonged collapse times significantly associated with large false aneurysms (≥3 cm) at the cardiac ends of the severed common carotid arteries. Seventy-one percent of cattle taking longer than 75 s to collapse exhibited large cardiac-end aneurysms (26).

Gibson (42) evaluated 644 cattle slaughtered without stunning under halal upright restraint, comparing low neck cuts (C2–C3, *n* = 561) with high neck cuts (C1, *n* = 83), and found that high neck cutting significantly reduced mean time to collapse (13.5 ± 1.3 s vs. 18.9 ± 1.1 s; *p* < 0.01). False aneurysm formation was more frequent and more severe in low neck cuts, with higher proportions of aneurysm scores ≥3, and animals taking >20 s to collapse were significantly associated with higher aneurysm scores, although aneurysm score alone did not independently predict collapse times ≥60 s (42). Bozzo (43) evaluated 1,200 cattle slaughtered without stunning by halal ventral neck incision at three anatomical levels (C1, C2, C3–C4) and reported an overall false aneurysm prevalence of 8.25%, with higher incidence at more caudal cutting sites (10.25% at C3–C4 vs. 7.25% at C1–C2). Approximately 37.5% of animals exhibiting signs of rhythmic breathing and corneal or palpebral reflexes at 90 s post-cut had false aneurysms (43).

### Computational modeling

Benchimol (27) used a numerical model of the bovine cerebrovascular system in COMSOL Multiphysics, a finite-element simulation software platform, to simulate bilateral carotid sectioning and subsequent hemodynamic changes. The model predicts a near-instantaneous (~99%) reduction in cerebral blood flow and pressure following carotid transection. Simulated post-incisional carotid occlusion scenarios have not been shown to restore or sustain cerebral blood flow or pressure (27). As a modeling study, these findings provide physiological plausibility rather than direct experimental evidence of consciousness or unconsciousness.

### Risk of bias

The overall risk of bias across the included studies in estimating the effect of carotid occlusion on outcomes related to inferred LOC was judged to be moderate. This assessment was driven principally by confounding and outcome-measurement limitations, including heterogeneity in animal age, stunning or anesthesia status, cervical cut location and procedural conditions, together with reliance on indirect behavioral and physiological proxies for LOC; intervention classification and documentation of slaughter conditions were generally less important sources of bias. The principal methodological limitations, particularly reliance on behavioral proxies such as collapse or reflex persistence, systemic hemodynamic measures as indirect surrogates for cerebral perfusion, and electrophysiological indices reflecting residual cortical electrical activity rather than integrated cortical function, constitute inherently indirect or delayed indicators of inferred LOC. Because these measures do not directly assess cortical perfusion or integrated cortical function, and because brainstem-mediated motor activity and residual electrical activities may persist after cortical awareness has already been lost, they may extend the apparent duration of consciousness. These limitations reduce the directness and certainty of inferences regarding LOC and preclude definitive conclusions based on any single proxy measure. A full ROBINS-I–based risk of bias assessment for all included studies is provided in Supplementary material 2.

### Certainty of evidence

Certainty of evidence was highest for the prevalence of carotid occlusion, moderate to low for hemodynamic effects, low for electrophysiological inference regarding LOC, low for behavioral indicators of inferred LOC, and low for computational modeling as direct evidence of consciousness. The overall certainty that CO delay inferred LOC was therefore judged low, primarily because no included study directly measured consciousness or cortical perfusion in relation to CO (Table 3).

## Discussion

This systematic review assesses the prevalence of CO and whether the available evidence suggests that CO following SEVNI have been shown to impact cerebral ischemia, cortical function, or the duration of consciousness. The included evidence base encompasses studies reporting associations between CO and prolonged collapse, persistent electrophysiological activity, increased residual jugular flow, and delayed decline in systemic arterial pressure, as well as studies that did not demonstrate such associations. It is important to acknowledge that no method during commercial slaughter directly measures cortical awareness or subjective experience in real time. Electrophysiology, hemodynamics, and behavioral indicators are therefore indirect proxies and must be interpreted cautiously. However, when multiple independent lines of evidence converge, meaningful physiological inference remains possible. In this context, the available evidence does not demonstrate that CO preserve cortical perfusion or integrated cortical function sufficiently to delay inferred LOC.

Across 15 included studies, CO, most commonly described as false aneurysms or arrested flow at the cardiac (caudal) ends of the severed common carotid arteries, were reported with variable frequency. For the descriptive prevalence calculation, five unstunned cohorts provided animal-level data suitable for inclusion (33, 34, 36, 37, 43), comprising 1,848 cattle. Using an inclusive definition encompassing false aneurysm formation, observed carotid occlusion, or arrested carotid blood flow, 255 of 1,848 animals (13.8%) exhibited at least one reported carotid occlusive phenomenon. Bilateral occlusions were considerably less common, typically below 8% of animals. The available prevalence studies therefore establish that carotid occlusions occur in a subset of cattle after SEVNI, but do not by themselves establish the physiological significance of these occlusions for cerebral perfusion. Supratikno et al. (37) reported false aneurysm formation only, rather than directly measuring carotid blood flow arrest, and therefore identify a structural phenomenon that may or may not result in functional occlusion. Rabkin (29) notes that the carotid occlusion hypothesis is speculative and predicated on largely observational data which cannot establish causality. In contrast, Rabkin (29) proposes an alternative, similarly plausible explanation, that CO arise secondary to the progressive reduction in blood flow that occurs during exsanguination (29). These considerations underscore the need to examine the physiological implications of carotid occlusion within the hemodynamic conditions that follow SEVNI.

Occlusion at the severed cardiac ends of the carotids is unlikely to recreate an intact pressurized arterial circuit. Rather, it occurs in the setting of an open (depressurized) system with ongoing systemic blood loss and rapidly declining arterial pressure. Bager et al. (38), in a hemodynamic study, reported that CO in two calves were associated with higher residual blood flow in the jugular veins but observed no clinical signs of return to consciousness, i.e., their behavior during exsanguination was similar to that of the calves without occlusions. This finding is consistent with the physiological principle that residual flow alone has not been shown to perfuse brain tissue in an open, rapidly depressurizing circulatory system.

The timing of occlusion formation must also be considered in the context of the rapid loss of circulating blood volume (hypovolemic shock) that follows exsanguination. Occlusions forming only after irreversible large volume blood loss has already occurred would not be expected to exert a physiologically meaningful effect on systemic or cerebral blood pressure. Notably, the only studies that quantified the time to initial carotid flow arrest reported a mean onset of approximately 20–24 s after incision (34, 39), by which point substantial blood loss would be expected to have occurred. Because restoration of cerebral perfusion requires an adequate upstream pressure gradient, occlusion of the severed cardiac ends at this late stage cannot itself recreate an intact, pressurized cerebral circulation. This temporal relationship is also compatible with the alternative hypothesis proposed by Rabkin (29), in which CO may arise secondarily as blood flow and arterial pressure progressively decline during exsanguination rather than serving as the primary cause of prolonged cerebral perfusion. The available evidence therefore indicates that carotid occlusions, when they occur, are first detected only after substantial blood loss, with no evidence that they form earlier or produce blood outflow interference sufficient to meaningfully alter cerebral perfusion or integrated cortical function.

In the bovine cerebrovascular model reported by Benchimol et al. (27), bilateral carotid transection was associated with an approximately 99% reduction in cerebral blood flow. In that model, simulated carotid occlusions, including scenarios with up to 80% obstruction of both the cephalad and cardiac ends of the severed vessels occurring immediately following the incision, did not restore cerebral perfusion pressure to levels that would be expected to support sustained cortical function (44–46). Importantly, these modeling data remain inferential and depend on several simplifying assumptions, including representation of vessels as nondeformable cylindrical conduits, simplified geometry of the rete mirabile and Circle of Willis, and prescribed inlet and outlet boundary conditions. The model also assumes continued cardiac output after carotid sectioning and does not reproduce the full dynamic cardiovascular response to progressive exsanguination. Notably, several modeling choices, including simplification of the rete mirabile and zero-relative-pressure outlet conditions, were intentionally conservative and were expected to overestimate rather than underestimate residual cerebral perfusion. The model should therefore be interpreted as supportive mechanistic evidence of physiological plausibility rather than as an empirical measurement of cerebral perfusion or consciousness *in vivo*.

Beyond these hemodynamic and temporal considerations, anatomical factors are also relevant to interpreting the carotid occlusion hypothesis of delayed LOC. Most studies described false aneurysms forming at the cardiac ends of the severed common carotid arteries. Therefore, even if clot formation restricts outflow at these sites, the vertebral–occipital anastomosis and the rete mirabile continue to alter circulation by providing alternative pathways that preferentially divert blood away from the cerebral arterial circle rather than toward it and flow out via the distal ends of the severed artery. Numerical modeling data support this interpretation, predicting that the hemodynamic state following bilateral carotid transection is dominated by flow reversal and rapid cerebral blood flow and pressure collapse rather than restoration of cerebral perfusion (27).

From an electrophysiological perspective, prolongation of the isoelectric point, HALF wave duration, or persistence of evoked potentials, as described by Bager et al. and Anil et al., reflect continued cortical electrical activity but do not provide evidence of preserved cortical function or conscious awareness (23, 39, 40, 47). In Bager et al. (39), transition to HALF activity occurred within approximately 10 s after incision and was interpreted as consistent with LOC, substantially preceding the eventual isoelectric state. Likewise, persistence of an evoked potential demonstrates residual electrophysiological responsiveness within portions of the sensory pathway but does not establish the integrated cortical network activity required for conscious sensory perception. Conscious awareness is a systems-level phenomenon that depends on integrated cortical network activity supported by adequate cerebral perfusion (48, 49). Accordingly, once cerebral perfusion falls below critical thresholds (44–46), consciousness is lost even if some residual blood flow or cortical electrical activity persists.

Behavioral indicators can be useful for animal welfare monitoring, particularly for practical slaughterhouse decision-making, but they are imprecise measures of the timing of LOC and should be interpreted cautiously (10, 11). Gregory et al. (26) and Gibson et al. (42) reported behavioral observations suggesting that a minority of cattle slaughtered without stunning remained standing for prolonged periods after the neck incision, with approximately 8% of animals in Gregory et al. requiring ≥60 s to reach final collapse and a small proportion in Gibson et al. also exhibiting delayed collapse times, findings that were reported to correlate with the presence or severity of CO. However, these observations warrant careful neuroanatomical interpretation for several reasons. First, relative to humans, motor output in domestic species relies far more heavily on extrapyramidal and brainstem circuits rather than cortical control. In bovines, maintenance of posture is mediated primarily by brainstem extrapyramidal pathways, particularly the reticulospinal and vestibulospinal tracts, such that persistence of posture or reflexive movements after cortical LOC is expected and is not, by itself, reliable evidence of preserved consciousness (50, 51). Second, there is a well-described cortico-brainstem dissociation in susceptibility to ischemia: cortical neurons are particularly vulnerable to acute reductions in oxygen delivery, whereas brainstem structures demonstrate greater hypoxic–ischemic resistance and may continue to support postural tone, rhythmic respiratory activity, and brainstem reflexes after cortical function has been lost (52, 53). Thus, delayed collapse, rhythmic breathing, and persistence of brainstem reflexes may systematically outlast cortical LOC and should not, in isolation, be interpreted as evidence of preserved conscious awareness.

This review is limited by heterogeneity in study design, slaughter method, animal age, anesthesia or stunning use, and outcome measures, precluding meta-analysis. No study directly measured cortical perfusion or integrated cortical function, instead relying on indirect proxies such as systemic blood pressure, jugular flow, isoelectric point on ECoG, evoked potentials, collapse time, or reflexes. These measures are imperfect indicators of consciousness, and behavioral endpoints lag behind cortical loss due to brainstem hypoxia resistance. Several electrophysiological studies were performed under anesthesia or stunning, potentially altering physiological responses. Many conclusions regarding delayed LOC were inferential rather than based on direct perfusion data, and publication bias cannot be excluded. Additionally, direct measurements of cortical perfusion during slaughter without stunning remain limited, and further physiological investigation could help clarify the precise temporal relationship between exsanguination, carotid occlusion, and LOC. Finally, because interpretation of indirect proxy measures is inherently vulnerable to investigator judgment, conclusions were revised to distinguish direct observations from physiological inference.

In summary, using an inclusive definition encompassing false aneurysm formation, observed carotid occlusion, or arrested carotid blood flow, 13.8% of animals in the eligible unstunned prevalence cohorts exhibited at least one reported carotid occlusive phenomenon. Despite this cumulative incidence, the available anatomical, hemodynamic, electrophysiological, behavioral, and numerical modeling data have not demonstrated that CO delay cerebral ischemia, preserve cortical function, or produce findings that can be reliably interpreted as delayed LOC. Therefore, based on available data, the CO hypothesis remains physiologically unproven. When occlusions do occur, the available timing data suggest that they arise after substantial blood loss has occurred, and there is currently no evidence to suggest that they can restore or sustain cerebral perfusion at levels compatible with consciousness.

## Data Availability

The original contributions presented in the study are included in the article/Supplementary material, further inquiries can be directed to the corresponding authors.
