# Supplementary material for: Common carotid artery occlusion during slaughter without stunning in cattle: incidence and evidence for physiological significance—a systematic review
Source: Front Vet Sci. 2026 Sep 15;13:1868851. doi: 10.3389/fvets.2026.1868851 (PMC13619484; doi:10.3389/fvets.2026.1868851)
Supplement: Supplementary file 1 [file Table_1.DOCX]

**Supplementary Material 2**

**ROBINS-I Risk of Bias Assessment**

Risk of bias for all 15 included studies in this systematic review was assessed using the ROBINS-I tool(Sterne et al., 2016), adapted for experimental, observational, electrophysiological, hemodynamic, and numerical modeling designs. Each study was evaluated across seven domains: confounding; participant selection; classification of interventions; deviations from intended interventions; missing data; outcome measurement; and selective reporting.

**1. Bias Due to Confounding**

Overall judgment: **Moderate to Serious**

No included study employed randomized allocation. Important potential confounders included cut position (C1 vs C2–C4), stunning status, anesthesia, restraint method (upright vs inverted), age (calf vs adult), and baseline cardiovascular status. Prevalence and behavioral field studies generally did not adjust for these variables. Experimental physiology studies controlled some parameters but introduced anesthesia-related confounding. Numerical modeling eliminated biological confounding but depends on structural assumptions. Confounding represented the dominant source of bias across the body of evidence.

**2. Bias in Selection of Participants**

Overall judgment: **Moderate**

Most animals were enrolled opportunistically in commercial slaughter settings or laboratory convenience samples. Inclusion and exclusion criteria were rarely prespecified. There was no clear evidence of systematic exclusion of unfavorable cases, but non-random sampling limits generalizability.

**3. Bias in Classification of Interventions**

Overall judgment: **Low**

Slaughter method, cut location, stunning status, and presence of carotid occlusion (false aneurysm or arrested flow) were clearly defined in all studies. Occlusion was typically identified by direct visual inspection, palpation, histology, or flow assessment. Misclassification risk was low.

**4. Bias Due to Deviations from Intended Interventions**

Overall judgment: **Moderate**

Procedural variability in commercial slaughter (cut precision, restraint conditions) could influence occlusion formation and bleeding dynamics. Experimental studies introduced additional deviations (e.g., anesthesia, post-incisional stunning). Formal protocol adherence monitoring was rarely reported.

**5. Bias Due to Missing Data**

Overall judgment: **Low to Moderate**

Large prevalence studies reported complete denominators with minimal attrition. Smaller electrophysiology studies occasionally lacked detailed individual-level reporting. No evidence suggested systematic missingness affecting direction of results.

**6. Bias in Measurement of Outcomes**

Overall judgment: **Moderate (physiological outcomes); Serious (behavioral proxies)**

Objective physiological measures (arterial pressure, ECoG/EEG patterns, jugular flow, numerical modeling outputs) were directly recorded and reproducible, although interpretation of electrophysiological endpoints (e.g., HALF activity, isoelectricity, evoked potentials) varied. Behavioral outcomes—particularly time to collapse or reflex persistence—were used as proxies for cortical loss of consciousness and are indirect, brainstem-mediated markers. These introduce substantial measurement limitations when interpreted as indicators of awareness.

**7. Bias in Selection of Reported Results**

Overall judgment: **Moderate**

Most studies predated trial preregistration and prespecified protocols. Selective emphasis on collapse times or aneurysm correlations was possible in some observational reports. However, there was no evidence of critical selective reporting or outcome suppression.

**Overall Risk of Bias Judgment**

No study met criteria for low risk of bias. Most studies were judged **moderate overall**, with behavioral collapse studies reaching **moderate to serious risk** primarily due to outcome measurement limitations. No study was considered at critical risk.

Across the body of evidence, bias sources were structural and methodological rather than directional. Importantly, reliance on delayed or indirect proxies (e.g., collapse) would be more likely to overestimate persistence of consciousness rather than obscure rapid cortical perfusion collapse.

**Overall ROBINS-I assessment for the included literature: Moderate risk of bias.**

Sterne, J.A., Hernán, M.A., Reeves, B.C., Savović, J., Berkman, N.D., Viswanathan, M., Henry, D., Altman, D.G., Ansari, M.T., and Boutron, I. (2016). ROBINS-I: a tool for assessing risk of bias in non-randomised studies of interventions. *bmj* 355.
