# Supplementary material for: Common carotid artery occlusion during slaughter without stunning in cattle: incidence and evidence for physiological significance—a systematic review
Source: Front Vet Sci. 2026 Sep 15;13:1868851. doi: 10.3389/fvets.2026.1868851 (PMC13619484; doi:10.3389/fvets.2026.1868851)
Supplement: Supplementary file 2 [file Table_2.DOCX]

**Supplementary Material:

Detailed Search Strategy for Carotid Occlusion Review**

**Databases and Search Dates**

A comprehensive literature search was conducted from database inception through February 10, 2026, across the following electronic databases: PubMed (MEDLINE), Web of Science Core Collection, Cochrane Library, MEDLINE (alternative interfaces), and Google Scholar. All searches were last updated on February 10, 2026.

No lower date limit was applied in order to capture early experimental and physiological studies, including foundational publications from the early and mid-20th century. No language restrictions were applied at the search stage.

**Search Strategy and Terms**

Search strategies were adapted to the indexing structure and syntax of each database. Controlled vocabulary (where available) and free-text terms were used. Synonyms and plural variants were incorporated to maximize sensitivity. Manual reference screening and citation chaining were performed to identify additional relevant studies.

**PubMed (MEDLINE)**

Primary search:

(calf OR bovine OR cattle)
AND
("carotid occlusion" OR "carotid occlusions" OR "false aneurysm" OR "false aneurysms")
AND
("blood flow" OR "blood pressure")
AND
slaughter
AND
("cerebral perfusion" OR "cerebral circulation" OR "cortical function")

Religious slaughter–specific PubMed search:

("carotid occlusion" OR "carotid occlusions" OR "false aneurysm" OR "false aneurysms")
AND
(calf OR bovine OR cattle)
AND
"blood pressure"
AND
(shechita OR halal)
AND
("cerebral perfusion" OR "cerebral circulation" OR "cortical function")

**Web of Science Core Collection**

TS=(calf OR bovine OR cattle)
AND
TS=("carotid occlusion" OR "carotid occlusions" OR "false aneurysm" OR "false aneurysms")
AND
TS=("blood flow" OR "blood pressure")
AND
TS=(slaughter OR bleeding OR shechita OR halal)
AND
TS=("cerebral perfusion" OR "cerebral circulation" OR "cortical function")

**MEDLINE (Alternative Interfaces)**

bovine AND carotid occlusion AND cerebral perfusion

Additional iterative searches incorporated combinations of:

false aneurysm AND bovine AND blood pressure
carotid occlusion AND cattle AND slaughter

**Cochrane Library**

(calf OR bovine OR cattle)
AND
("carotid occlusion" OR "false aneurysm")
AND
("cerebral perfusion" OR "cerebral circulation")

**Google Scholar**

Due to limited Boolean precision, iterative keyword searches were conducted using combinations of the following terms:

- bovine carotid occlusion cerebral perfusion
- cattle false aneurysm blood pressure slaughter
- carotid occlusion cattle shechita
- bovine false aneurysm halal slaughter
- carotid occlusion cerebral circulation cattle

For each query, the first 200 results sorted by relevance were screened. Citation chaining was used to identify additional historical and foundational studies.

**Filters and Eligibility Criteria**

- **Species:** Cattle (Bos taurus)
- **Study types:** Experimental, observational, anatomical, physiological, or hemodynamic studies
- **Exclusions:** Reviews without original data, editorials, opinion pieces, conference abstracts without full text
- **Time limits:** None applied

**Study Selection and Data Extraction**

Titles and abstracts were independently screened by co-authors. Full texts of potentially eligible studies were reviewed for inclusion. Disagreements were resolved by consensus. Reference lists of included articles were manually screened for additional relevant publications.

Extracted data included:

- Authors and year of publication
- Study design
- Slaughter or experimental method
- Presence and characterization of carotid occlusion or false aneurysm
- Hemodynamic measurements
- Indicators of cerebral perfusion or cortical function
- Key findings

**Synthesis**

Given heterogeneity in study design, outcome measures, and historical methodology, results were synthesized narratively. Quantitative pooling and meta-analysis were not performed. Reporting followed PRISMA guidelines where applicable.
